# Potential Glycobiomarkers in Maternal Obesity and Gestational Diabetes During Human Pregnancy

**DOI:** 10.3390/jcm14051626

**Published:** 2025-02-27

**Authors:** Anna Farkas, Andrea Suranyi, Balint Kolcsar, Zita Gyurkovits, Zoltan Kozinszky, Sandor G. Vari, Andras Guttman

**Affiliations:** 1 Horváth Csaba Memorial Laboratory of Bioseparation Sciences, Research Center for Molecular Medicine, Faculty of Medicine, University of Debrecen, H-4032 Debrecen, Hungary; anna.farkas@med.unideb.hu (A.F.); guttmanandras@med.unideb.hu (A.G.); 2 Department of Obstetrics and Gynecology, University of Szeged, H-6725 Szeged, Hungary; kolcsar.balint@med.u-szeged.hu (B.K.); gyurkovits.zita@med.u-szeged.hu (Z.G.); kozinszky@gmail.com (Z.K.); 3 Capio Specialized Center for Gynecology, Solna, 18288 Stockholm, Sweden; 4 Research and Innovation in Medicine Program, Cedars-Sinai Medical Center, Los Angeles, CA 90048, USA; sandor.vari@cshs.org; 5 Translational Glycomics Group, Research Institute of Biomolecular and Chemical Engineering, University of Pannonia, Egyetem u 10, H-8200 Veszprem, Hungary

**Keywords:** capillary electrophoresis, gestational diabetes mellitus, human IgG N-glycome, maternal obesity, molecular diagnostics

## Abstract

**Introduction:** Obesity is a rapidly growing common health problem worldwide that can lead to the development of gestational diabetes mellitus (GDM). However, GDM not only affects women with obesity but can also develop at any time, even after the OGTT test; therefore, an increasing number of complications related to GDM can be seen in both mothers and their children. It is necessary to discover biomarkers capable of indicating the development of GDM or complications during/after pregnancy. Since the N-glycosylation motif of human IgG has been described to change under many physiological and pathological conditions, it is a promising target for biomarker research. In our study, the effects of obesity and GDM were investigated on human serum IgG N-linked glycosylation patterns during human pregnancy. **Materials and Methods:** The study participants were categorized into four groups according to their body mass index (BMI) and GDM status: normal weight as control, obese (BMI > 30 kg/m^2^), normal weight with GDM, and obese with GDM. The released N-glycan components of IgG were separated with capillary electrophoresis and detected using a laser-induced fluorescence detector. **Results:** The result revealed several differences between the N-glycosylation patterns of the four study groups. Of this, 17 of the 20 identified structures differed significantly between the groups. The ratios of sialylated to non-sialylated structures were not changed significantly, but the core fucosylation level showed a significant decrease in the GDM and obese GDM groups compared to the control subjects. The lowest degree of core fucosylation was observed in the GDM group. **Conclusions:** The findings indicate that obesity in isolation does not have a significant impact on the IgG N-glycosylation pattern in pregnancy. Conversely, alterations in the N-glycan profile of antibodies may serve as biomarkers for the diagnosis of GDM in mothers with a normal BMI, although more evidence is needed. By incorporating glycan-based biomarkers into clinical practice, healthcare providers can improve early detection, personalize management strategies, and potentially mitigate adverse pregnancy outcomes associated with obesity and GDM.

## 1. Introduction

Gestational diabetes mellitus (GDM) is a type of diabetes diagnosed during pregnancy. GDM is attributed to high blood glucose levels during pregnancy in women with no previous history of impaired insulin sensitivity. This condition may develop because pregnancy hormones cause cells to use insulin—the hormone that regulates blood glucose levels—less efficiently [1]. GDM occurs in about 10% of all pregnancies in Europe [1,2], making it the most common medical complication during pregnancy [1,2,3]. In recent decades, an increasing prevalence of GDM has been reported worldwide. The overall international standardized prevalence of GDM was 14.0%. The regional standardized prevalence of GDM was significantly different: 7.8% in Europe and 27.6% in the Middle East [3,4].

GDM and obesity are closely interrelated conditions that pose significant health risks to both mothers and newborns. The prevalence of maternal obesity is rising rapidly worldwide, making it a critical obstetric risk factor. Similarly, the incidence of GDM is increasing across all continents, reflecting broader global health trends. Of particular concern is the growing prevalence of obesity among women of childbearing age, which further exacerbates the risk of GDM [3,4]. During pregnancy, women typically exhibit higher levels of galactosylation and sialylation of immunoglobulin G (IgG) glycans. These modifications are essential components of glycosylation processes that occur in response to the physiological and immunological adaptations necessary for a successful pregnancy [5]. Increased galactosylation of IgG contributes to an anti-inflammatory immune profile, which plays a crucial role in maintaining maternal–fetal tolerance and preventing adverse immune responses [5,6]. Additionally, sialylation, the process of adding sialic acid residues to glycoproteins, also increases during pregnancy [5,6]. This modification further enhances the anti-inflammatory effects and strengthens the overall immunomodulatory environment [6]. By promoting immune tolerance toward the fetus while preserving maternal immunity, these glycosylation changes ensure a balanced and protective immune response throughout pregnancy [5,6,7].

Various pathological glucose metabolism conditions can have a direct impact on galactosylation and sialylation. Human IgG glycosylation is currently at the forefront of both immunology and glycobiology [8]. For decades, it has been recognized that the conserved N-linked glycan on asparagine 297 found within the second Ig domain of the heavy chain that helps to comprise the Fc region of IgG plays a special role in IgG structure and function [8]. Changes in galactosylation, fucosylation, and sialylation are now well-established factors that drive differential IgG function, ranging from inhibitory and anti-inflammatory to activating complement and promoting antibody-dependent cellular cytotoxicity [8].

Change reveals to analyze the effect of overweight on IgG galactosylation and sialylation during human pregnancy complicated by obesity and/or GDM. We would like to find a new biomarker for screening the pregnant population at high risk for obesity in order to prevent complications of GDM and obesity.

## 2. Materials and Methods

### 2.1. Reagents and Buffers

Overall, 1000+ PhyTip chromatography microcolumns with 40 µL Protein G bed volume and the Conditioning and Capture buffers were purchased from PhyNexus Inc. (San Jose, CA, USA). A total of 10 kDa spin filters with polyethersulfone (PES) membrane and sodium dodecyl sulfate (SDS) were from VWR (Radnor, PA, USA). Sodium cyanoborohydride (NaBH_3_CN, 1M in tetrahydrofuran), dithiothreitol (DTT), and NaHCO_3_ were obtained from Sigma Aldrich (St. Louis, MO, USA). Peptid-N-glycosidase F enzyme (PNGase F) used to liberate the N-glycan content of the purified IgGs was ordered from Asparia Glycomics (San Sebastián, Spain). The Fast Glycan Labeling and Analysis Kit, including the 8-aminopyrene-1,3,6-trisulfonic acid (APTS) labeling dye, the magnetic beads, maltooligosaccharide ladder, and maltose as standard, as well as the N-linked carbohydrate separation gel buffer (NCHO), was purchased from Bio-Science Kft. (Budapest, Hungary). The bare fused silica capillary with a 50 µm inner diameter was from Optronis GmbH. (Kehl, Germany). Acetic acid was purchased from MOLAR CHEMICAL Kft. (Halasztelek, Hungary).

### 2.2. Samples

A prospective, cross-sectional cohort study was conducted on pregnant women undergoing delivery at the Department of Obstetrics and Gynecology, University of Szeged, Hungary. The timing of serum sample collection was 7 months, from January 2019. The 34 + 0 and 40 + 6 weeks of gestation were recruited into our study. Exclusion criteria of the study were identified as follows: multiple pregnancies; fetal or neonatal structural or genetic anomaly; improper localization of the placenta (e.g., placenta praevia); pathological placentation (placenta accreta spectrum); or self-reported drug, alcohol, or nicotine abuse.

The determination of gestational age was based on the first day of the last menstrual period and/or on ultrasound biometry (crown-rump-length and biparietal diameter) at the 10th week of pregnancy.

The serum samples collection—from 20 normal pregnant women, 20 pregnant women with obesity (BMI > 30 kg/m^2^), 7 pregnant women with gestational diabetes, and 5 pregnant women with gestational diabetes and obesity—were carried out at the Obstetrics and Gynecology Clinic, University of Szeged.

The GDM diagnostic criteria used were based on the World Health Organization (WHO) guidelines [9] presented in Table 1.

High-risk pregnancies for GDM [10] were screened with OGTT between 12 and 16 weeks of gestation, and if the result was below the limit, OGTT was repeated between 24 and 28 weeks of gestation. In our country, patients who do not have a high risk for GDM are screened with OGTT when they are 24–28 weeks pregnant. The characteristics of the participants involved in the study are summarized in Table 2.

We collected venous blood sampling from pregnant women (500 µL) with the written consent of the patient. Data collection was prospective and retrospective from medical history.

The blood samples were collected into a 5 mL CAT serum vacuette tube (Greiner Bio-One GmbH, production location: Greiner Bio-One Thailand Ltd. Chonburi, Thailand), centrifuged at 1500× *g* for 10 min at 5 °C. Separated serum samples were stored in 1.5 mL Eppendorf tubes (Dialab Kft., Budapest, Hungary) in an Arctiko ULUF 490-2M ultra-low temperature freezer (Arctiko Ltd., Salisbury, UK) at −80 °C until analysis.

### 2.3. IgG Purification and Preparation for N-Glycan Analysis by CE-LIF

IgG antibodies of maternal serum samples were purified by 1000+ PhyTip chromatography microcolumns with 40 μL Protein G bed volume as described in our previous publication with some modifications [11]. Briefly, 200 µL serum samples were diluted with 200 µL Capture/Wash 1 Buffer (10 mM NaH_2_PO_4_ and 0.14 M NaCl, pH 7.4). The columns were conditioned before the purification step using 500 µL of Capture/Wash 1 Buffer. The captured antibodies were eluted from the columns with 200 µL acetic acid (10%). Between the capture and elution steps, the columns were washed once with the Capture/Wash 1 Buffer and once with Wash 2 Buffer (0.14 M NaCl).

The denaturation, reduction, and release of asparagine-linked glycan structures from the captured IgG molecules were carried out on 10 kDa spin filters. After the elution buffer was removed by centrifugation (11,384× *g* for 10 min.), 70 µL water was added to the samples and centrifuged again (11,384× *g* for 10 min) to completely remove the acetic acid. For protein denaturation, a 14 µL denaturation mixture (10 µL water, 4 µL 400 mM DTT, and 5% SDS) was added to the samples and incubated at 65 °C for 10 min. The N-linked oligosaccharides were enzymatically removed using 1 µL PNGase F enzyme (200 mU) dissolved in 49 µL of 20 mM NaHCO_3_ (pH 7.0). The liberated N-glycans were separated from the polypeptide backbone by centrifugation at 11,384× *g* for 10 min, dried in a vacuum centrifuge, and labeled at 37 °C overnight using 6 µL 20 mM APTS (in 15% acetic acid) and 2 µL sodium cyanoborohydride (1M in tetrahydrofuran). The excess labeling dye was removed by using magnetic beads; then, the samples were analyzed by capillary electrophoresis with laser-induced fluorescence detection (CE-LIF) or stored at −20 °C until analysis.

### 2.4. CE-LIF Analysis

Capillary electrophoretic analysis of the liberated N-linked oligosaccharides from the IgG antibodies purified from the serum samples of the mothers of the investigated groups was accomplished by using a PA800 ProteomeLab system equipped with a laser-induced fluorescent detector (excitation: 405 nm; emission: 520 nm) (Beckman Coulter Inc., Brea, CA, USA). For the separations, a bare fused silica capillary with 50 cm effective and 60 cm total length (50 µm inner diameter) was filled with NCHO separation gel buffer. The separations were carried out at 25 °C cartridge temperature using a 30 kV applied voltage (cathode at the injection side, anode at the detection side). To calculate the GU values of the separated carbohydrate peaks for accurate structure determination, an APTS-tagged maltooligosaccharide ladder was run with each sample set. Before the sample injection, APTS-labeled maltose internal standard was pressure injected by applying 1 psi (6.89 kPa) for 5 s [12].

### 2.5. Data and Statistical Analyses

During the separation of the N-glycan structures, data acquisitions were performed by the 32Karat (version 7.0) from Beckman Coulter. The GU value of any unknown structure can be determined by comparing the CE migration time of the peak of interest with the migration time of the corresponding peaks of a linear homooligosaccharide ladder consisting of α1-4 linked oligo-glucose units. The differences in the type of glycosidic bonds and the anomeric configuration of the oligosaccharides are crucial in determining the hydrodynamic volume. Consequently, these differences can significantly influence the migration time of the isomers. By using a suitable internal standard, structural assignments based on the GU value result in high accuracy. According to this approach, the N-glycan structures of interest were assigned to the detected peaks based on the GU values calculated with the GUcal software (v11b) (University of Pannonia; Veszprém, Hungary, https://mybiosoftware.com/tag/gucal). In addition to the built-in database in the software, the exact glycan structures in the profile were also clarified using literature data. PeakFit software (v4.12, SeaSolve Software Inc., San Jose, CA, USA) was used to determine the relative area percentages of the peaks. The values obtained as the results of the glycosylation analyses are shown as mean ± standard deviation (SD) in the tables and figures of this publication.

Since the Shapiro–Wilks test indicates that the datasets do not follow the normality, Kruskal–Wallis tests were employed for analyses of the continuous variables of N-glycosylation. The differences between the groups were considered significant at *p* ≤ 0.05.The demographic data, such as maternal age, BMI, and gestational age, were evaluated with a one-way ANOVA test (Dunett’s post hoc test). The data on weight gain was performed using an unpaired *t*-test. Significance was accepted at *p* < 0.05.

## 3. Results

### 3.1. Demographical Data

In our obese group, the mean maternal age (29.0 years) was not significantly different, while the maternal age in group GDM (34.5 years) and the group GDM combined with obesity (34.0 years) were significantly higher compared to the national average reference age at delivery, which was 28.86 years for primiparous women and 30.34 years for the total number of pregnant women in 2019 [13]. In the normal weighted group, as a control group we recruited pregnant women with similar demographical data as the national average. Women with obesity who participated in our study had a median BMI of 34.96 kg/m^2^, 24.4 kg/m^2^ in the GDM group, and 41.4 kg/m^2^ in the obese with GDM group at the first medical visit. The mean BMI of the normal weight group was 23.2 kg/m^2^. The weight gain during pregnancy was more pronounced among normal-weight pregnant women. By the end of pregnancy, the women in the control group gained significantly more weight (>80%) than their counterparts categorized into the other three groups. Demographic data are shown in Table 2.

### 3.2. IgG N-Glycosylation Analysis

The aim of the N-glycosylation analysis was to identify possible carbohydrate modifications of the IgG molecules obtained from the serum samples of the mothers after childbirth. Revealing these alterations may be potentially useful for early identification of the harmful effects of obesity and GDM during pregnancy.

**Figure 1:**
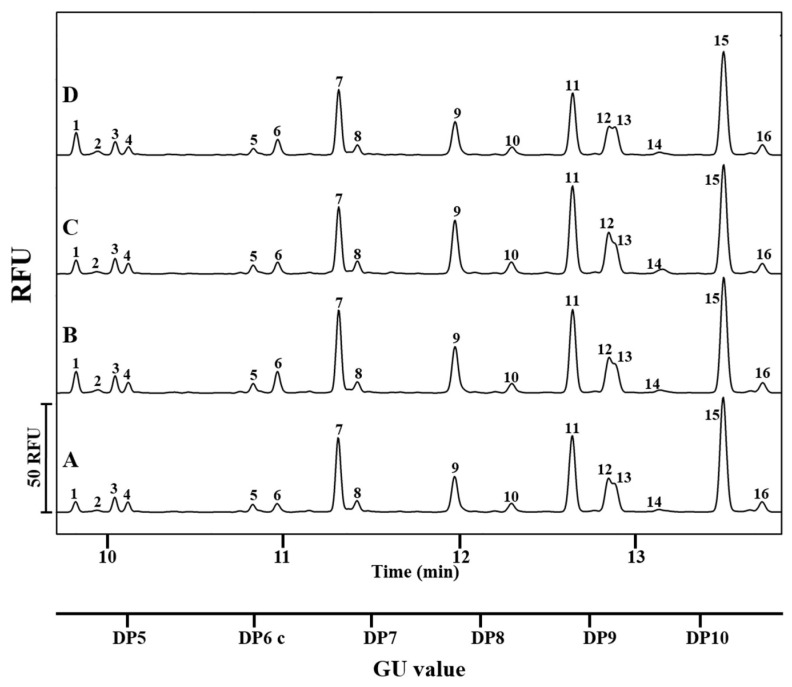
N-glycosylation patterns of IgG partitioned from serum samples of (**A**) normal weight (*n* = 20), (**B**) obese (*n* = 20), (**C**) GDM (*n* = 7), and (**D**) obese and GDM (*n* = 5) maternal groups analyzed by CE-LIF. The migration time of the peaks is shown in minutes on the upper X-axis, while the lower x-axis represents the GU values. The samples of normal weight and obese groups were pooled before the IgG purification step. Detailed structure and area percentage data of the peaks are listed in Table 3. Conditions: 60 cm total length (50 cm effective length) bare fused silica capillary (BFS) filled with NCHO separation gel buffer was used to separate the APTS labeled N-glycan components. Samples and maltose were injected into the capillary with 1 psi pressure for 5 s. The separations were accomplished by applying 30 kV at 25 °C temperature.

During the analysis of the asparagine-bound glycan profiles of maternal IgG isolated from the serum samples of normal weight, obese, GDM, and obese GDM groups, 16 peaks were detected. Based on their GU values and previously published data [11,14], 20 structures were identified, of which 4 co-migrated with other structures during the separation. The GU values of the peaks, the identified N-glycan structures, and their relative area percentage values are listed in Table 3. The representative N-glycan patterns of the investigated maternal groups are shown in Figure 1. The most identified structures were core-fucosylated two-antennary glycans.

In the electropherograms, the first 8 peaks represent N-glycan structures terminated with 1 or 2 sialic acids, which are of great importance in mediating the anti-inflammatory functions of the IgG antibodies. Non-sialylated, neutral structures were found between peaks 9 and 16. Core-fucosylated glycans responsible for the ADCC function accounted for more than 70% of the total carbohydrate profiles for all groups.

**Figure 2:**
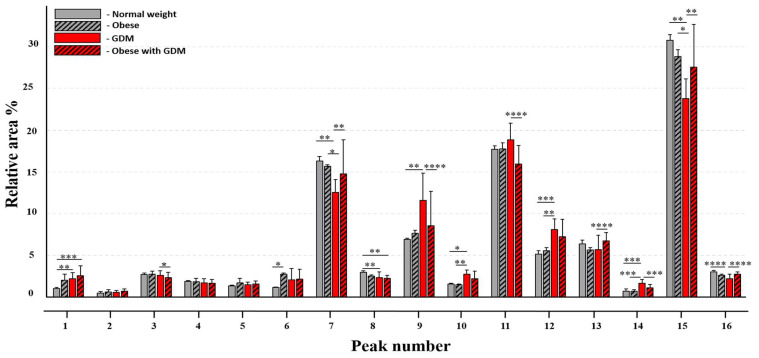
Significant differences between peaks representing IgG N-glycan structures of normal weight (gray; *n* = 20), obese (diagonally gray striped; *n* = 20), GDM (red; *n* = 7), and obese with GDM (red diagonal striped; *n* = 5) maternal groups. (* *p* ≤ 0.05, ** *p* ≤ 0.01, *** *p* ≤ 0.001, **** *p* ≤ 0.0001). The x-axis of the figure shows the peak number of the identified N-glycan structures; the y-axis shows the relative area percentage values of the peaks; the error bars represent the standard deviation of the dataset. The statistical analysis was performed using the Kruskal–Wallis test and Dunn’s post hoc test (* *p* ≤ 0.05, ** *p* ≤ 0.01, *** *p* ≤ 0.001, **** *p* ≤ 0.0001).

In the next stage of the study, the obtained IgG N-glycan profiles of the investigated maternal groups were subjected to statistical analysis to identify the N-glycosylation differences among the groups. The analysis revealed several alterations, which are detailed below and plotted in Figure 2.

As a result of the analysis with the Kruskal–Wallis test and Dunn’s post hoc test, the relative area percentage value of peak #6 corresponding to the N-glycan structure of A2G2S1 (*p* = 0.0208) showed a significant decrease in the control group compared to the obese group.

Comparing the control group to the GDM group, a significant difference was found in the area percentage value of nine peaks (peaks #1, #7, #8, #9, #10, #12, #14, #15, and #16). Among the sialic acid terminated structures, remarkable changes were found in peaks #7 (FA2G2S1; *p* = 0.0054) and #8 (FA2BG2S1; *p* = 0.0047), showing a decrease, while the relative area percentage of peak #1 (A2G2S2; *p* = 0.0059) increased in the case of the GDM group compared to the control. Among the neutral structures, peaks #9 (FA2, A2[6]G1; *p* = 0.0041), #10 (FA2B, A2B[6]G1; *p* = 0.0204), #12 (FA2[3]G1; *p* = 0.0006) and #14 (FA2B[3]G1, A2BG2; *p* = 0.0002) showed an increase in the N-glycosylation profile of IgG isolated from the serum samples of the GDM mothers compared to the control group. A decrease was identified in the area percentage values of peaks #15 and #16 corresponding to the core-fucosylated biantennary bigalactosylated (FA2G2; *p* = 0.0029) and core-fucosylated biantennary bisecting bigalactosylated (FA2BG2; *p* ≤ 0.0001) structures in the case of the GDM group compared to the control.

The obese GDM group could be differentiated from the control maternal group based on the relative area percentage values of two structures terminated by sialic acids. Peak #1 (A2G2S2; *p* = 0.0005) was increased, while peak #8 (FA2BG2S1; *p* = 0.0025) was decreased compared to the group of normal-weight healthy mothers.

We also found differences between the obese and GDM groups where an increase in the area percentage of peaks #10 (FA2B, A2B[6]G1; *p* = 0.0089), #12 (FA2[3]G1; *p* = 0.0082) and #14 (FA2B[3]G1, A2BG2; *p* = 0.0001) was observed in the GDM group compared to the obese. On the contrary, the peak area percent values of peaks #7 (FA2G2S1; *p* = 0.0318) and #15 (FA2G2; *p* = 0.0193) decreased in the GDM group compared to the obese group.

Interestingly, no significant difference was found between the IgG N-glycosylation of obese and obese GDM groups.

On the other hand, comparing the GDM to the obese GDM group, eight peaks showed notable differences. The relative area percentage value of peaks #3 (FA2G2S2; *p* = 0.0151), #9 (FA2, A2[6]G1; *p* ≤ 0.0001), #11 (FA2[6]G1; *p* ≤ 0.0001) and #14 (FA2B[3]G1, A2BG2; *p* ≤ 0.0001) decreased in the obese GDM group. An increase was observed, on the other hand, in the calculated area percentage of peaks #7 (FA2G2S1; *p* = 0.0074), #13 (FA2B[6]G1, A2G2; *p* ≤ 0.0001), #15 (FA2G2; *p* = 0.0012) and #16 (FA2BG2; *p* ≤ 0.0001). The statistically significant alterations between the investigated maternal groups are presented with the corresponding *p* values in Figure 2. The *p* values obtained as a result of the statistical analysis are shown in Table 4.

### 3.3. Calculation of the Sialo Form/Neutral Form (SF/NF) and the Core Fucosylation/Total N-Glycosylation (CF/TNG) Ratios

In order to evaluate the N-glycosylation properties affecting the immune functions related to IgG antibodies, we determined the ratio of sialylated structures to neutral structures belonging to each study group, as well as the degree of core fucosylation.

Interestingly, the ratio of sialylated structures showed a slight increase in the obese (0.43 ± 0.05) and obese with GDM groups (0.40 ± 0.12) compared to the control group (0.39 ± 0.01). On the other hand, the sialylation ratio calculated from the N-glycan profile of IgG isolated from the group of GDM mothers decreased (0.36 ± 0.09) compared to the control mothers. Although these changes are interesting, they were not significant according to the Kruskal–Wallis test.

**Figure 3:**
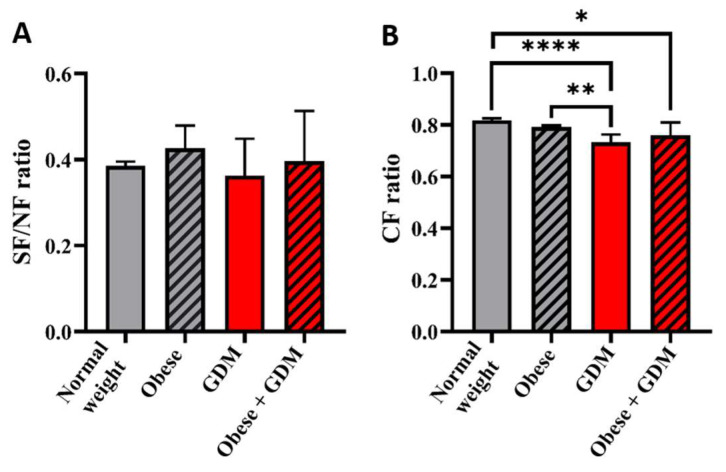
The SF/NF and CF/TNG ratio of maternal IgG N-glycan profile (case numbers: normal weight *n* = 20, pregnant women with obesity *n* = 20, pregnant women with gestational diabetes *n* = 7, and pregnant women with obesity *n* = 5). (* *p* ≤ 0.05, ** *p* ≤ 0.01, **** *p* ≤ 0.0001). The picture shows the statistical analysis result using the Kruskal–Wallis and Dunn post hoc test on the ratio of sialylated structures to non-sialylated (**A**) and the ratio of core fucosylation to total IgG N-glycome (**B**). The values are expressed as average values of the groups calculated from the CE-LIF analysis of all samples in the groups, and the error bars indicate the calculated standard deviations. The differences were considered significant if the *p* value was equal or less than 0.05. The significance level is indicated in the figure according to the obtained *p* value: * *p* ≤ 0.05, ** *p* ≤ 0.01, **** *p* ≤ 0.0001.

To determine the degree of core fucosylation, the sum of the area of all identified core-fucosylated structures was divided by the total glycosylation. The calculated core fucosylation level showed a slight but statistically significant decrease in the GDM (0.73 ± 0.030; *p* ≤ 0.0001) and obese GDM groups (0.76 ± 0.050; *p* = 0.0247) compared to the control subjects (0.81 ± 0.007). Although the level of core fucosylation decreased in the obese group (0.79 ± 0.005), the changes were insignificant compared to the control group (*p* > 0.9999). In the GDM group (0.73 ± 0.030), we observed the lowest degree of core fucosylation as its value was significantly lower compared to the control (0.81 ± 0.007), as well as to the mother cohort with obesity (0.79 ± 0.005, *p* = 0.0079). The results of the analysis are shown in Figure 3.

## 4. Discussion

Given the increasing prevalence of obesity, it has become a significant concern during pregnancy [16]. The high BMI before pregnancy is a considerable risk factor for the development of GDM [4,17].

The most important result of this pilot study was that the obese GDM group could be differentiated from the control maternal group based on the relative area percentage values of only one structure terminated by sialic acid (peak #6; A2G2S1). We also found differences between the obese and GDM groups where an increase in the area percentage of peaks #10 (FA2B, A2B[6]G1), #12 (FA2[3]G1), and #14 (FA2B[3]G1, A2BG2) was detected in the GDM group compared to the obese. On the contrary, the peak area percent values of peaks #7 (FA2G2S1) and #15 (FA2G2) decreased in the GDM group compared to the obese. On the other hand, comparing the GDM to the obese-GDM group, more peaks representing asparagine-linked glycan structures showed notable differences. The ratio of sialylated structures showed a slight increase in pregnant women with obesity, while the sialylation ratio in the group of GDM cases decreased compared to the normal-weighted pregnant women. The ratio of core-fucosylated N-glycan structures significantly decreased in the GDM and obese GDM groups compared to the control group. Furthermore, the level of core fucosylation of IgG isolated from the GDM group was significantly lower than that observed in the obese group.

The importance of early detection of GDM is widely recognized and an increasing number of studies are emerging focusing on promising diagnostic markers [3,16-17]. However, there is no commonly accepted criteria system for establishing such a diagnosis [3]. Previous research has indicated that changes in the N-glycosylation of proteins occur in various diseases [11,18,19,20,21,22]. Therefore, this study aimed to identify differential diagnostic markers associated with the development of GDM in women with both obesity and normal BMI. CE-LIF was used to analyze the APTS-labeled N-linked oligosaccharides of IgG obtained from serum, and differences were identified in the relative area percentage values of the N-glycan structures between the investigated groups.

While numerous alterations associated with obesity have been documented previously [23], we unexpectedly observed a difference in the relative area percentage of a single structure (A2G2S1) between the control and obese groups. In the obese group, the area percentage of the A2G2S1 structure increased more than twofold compared to the control group. However, it is important to note that the samples analyzed in prior studies included both sexes, with female participants not being pregnant at the time of sample collection. Thus, we can conclude that the changes in the serum IgG N-glycome during pregnancy may diminish the obesity-induced alterations that were observed in non-pregnant women with obesity.

The present research identified noteworthy modifying effects of GDM on IgG N-glycosylation, as 9 structures differed significantly between the control and GDM groups. These glycans may function as biomarkers for GDM in individuals with a normal BMI and assist in identifying pregnant women with an elevated risk for GDM despite maintaining a normal BMI. Weight gain during pregnancy was more favorable in the complicated groups (obese, GDM, and GDM–obese) compared to the normal group. Anyway, the weight gain stayed in the normal range in all study groups [24]. Furthermore, a substantial number of differing structures were observed between the GDM group and the group of participants with GDM and obesity. In instances where the same peaks demonstrated differences in comparisons between the control group and individuals with GDM, as well as between those with GDM and obesity-related GDM, the observed changes were relatively more moderate in the obese with GDM group compared to the control. This observation supports the prior conclusion that obesity exerts a lesser influence on the glycosylation profile in the subjects of this study and may mitigate some of the adverse effects associated with GDM.

As no significant differences were observed between the obese and obese with GDM groups, it can be concluded that the IgG N-glycan profile is not a valid biomarker for identifying GDM in obese pregnant women.

Previous studies have indicated a correlation between alterations in glucose and lipid metabolism and the onset of GDM. However, neither the overall N-glycosylation of plasma nor the N-glycan structures of IgA or IgG could effectively distinguish between the control and the GDM cohort. However, a more detailed N-glycosylation analysis of IgG revealed that FA2B and A2G2S2 structures showed a negative correlation with insulin levels and insulin sensitivity [25]. Here, we also observed the modification in the area percentage value of FA2B structure in both the GDM and OGDM groups; however, the increase was statistically significant only in the GDM group compared to the control. In contrast, the obese group demonstrated a slight decrease in FA2B levels relative to the control. This pattern was observed in both the obese and obese GDM groups, wherein the FA2B structure in individuals with obesity with GDM exhibited a lower area % value compared to those with GDM not associated with obesity. The increase in the relative area percentage of FA2B structure on IgG has also been noted in type 2 diabetes mellitus (T2DM) [26]. A characteristic phenomenon observed during pregnancy is impaired insulin sensitivity, which the pancreatic β cells attempt to compensate by increasing insulin secretion. If this compensation is insufficient, maternal hyperglycemia may develop, which is also observed in connection with GDM. The pathophysiology of GDM closely resembles that of type 2 diabetes. Consequently, the capacity for insulin secretion may play a more critical role in the development of GDM. Therefore, our observations regarding the FA2B N-glycan structure support the hypothesis that an increase in the ratio of the FA2B may be indicative of the development of insulin resistance [25,26].

N-glycosylation of IgG can reflect a change in pro- and anti-inflammatory properties, with one important marker being the level of sialylated N-glycans present in the molecules [23]. In the context of obesity, an increase in the amount of agalactosylated and bisecting GlcNAc structures and a decrease in sialylation have been reported [23,26]. Most of the observed differences were primarily related to central obesity rather than elevated BMI, as it is known that central obesity is mainly associated with subclinical inflammation [27]. Dietary modifications and bariatric surgery have been reported to reverse these changes, resulting increase in the level of sialylation and a decrease in agalactosylation, core fucosylation, and bicesting GlcNAc [28]. The literature data on the effects of weight loss on IgG N-glycosylation are not clear, as in a previously physically inactive obese group, weight loss following regular exercise was associated with an increase in the proportion of pro-inflammatory glycoforms. This observation can be attributed to the fact that the studied population was in the early stage of weight loss [29]. In our study, we specifically focused on the ratio of sialic acid-terminated structures to non-sialic acid-bearing structures. Although we observed a decrease in the ratio of sialylated structures in the GDM group compared to the control, this decrease was not significant. Interestingly, the degree of sialylation showed an increase in both the obese and obese GDM groups. The observed alterations in the SF/NF ratio were insignificant in any comparison. The results of Tanigaki et al. showed that hyposialylated IgG plays an important role in the reduction in insulin sensitivity associated with obesity and the consequent development of T2DM through the activation of the FcγIIB receptor [30]. Our results do not support the previous finding and may require further study with larger cohorts to gain a more comprehensive understanding of the role of sialylated serum IgG N-glycans in obesity and GDM.

Modifying the innermost GlcNAc in the N-glycan core structure of the IgG Fc region by incorporating fucose significantly enhances the affinity of IgG antibodies for the FcγIIIa receptor, thereby improving its capacity to induce antibody-dependent cellular cytotoxicity (ADCC) [31]. Examining core fucosylation, we observed a decrease in the fucosylation levels in all groups except for the obese compared to the control group. The findings of our study suggest that GDM has a more substantial impact on IgG core fucosylation than obesity. Moreover, a moderating effect of obesity can be observed for this glycosylation property. The observed change may indicate an increased ability of IgG to induce ADCC; however, additional research is necessary to assess whether and how these alterations influence immune functions under the conditions we investigated.

The results must be interpreted in light of the dynamic alterations in IgG N-glycosylation throughout pregnancy. Bondt et al. identified significant glycosylation modifications in both the Fc and Fab regions, with the Fab region displaying greater variability. They observed an increase in galactosylation and sialylation alongside a decrease in bisected GlcNAc and fucosylated structures relative to control subjects [32]. Considering these findings, the variations in sialylation and fucosylation should be regarded as normal physiological adaptations, which are particularly pronounced in individuals with obesity and GDM, especially concerning fucosylation. Thus, during pregnancy complicated by comorbidities, hormonal changes may diminish the distinctions between specific cohorts (in this case, obese and diabetic). These, in turn, may mask the differences in glycosylation profiles that arise due to underlying pathological changes.

All things considered, these findings suggest that obesity does not significantly influence the N-glycosylation pattern of IgG. However, alterations in the N-glycan profile of antibodies may serve as valuable indicators for diagnosing GDM in mothers with a normal BMI. Furthermore, these variations could help to pinpoint individuals within the normal BMI range at an increased risk of developing GDM.

These findings suggest potential clinical applications in identifying and differentiating pregnant women at risk for obesity-related GDM based on glycan profiling. Specifically, the distinct glycosylation patterns could serve as biomarkers for early diagnosis and risk stratification in prenatal care. In first-trimester screening, glycan profiling could help stratify pregnant women with obesity based on high metabolic risk. Pregnant women with glycosylation patterns indicating a higher risk of GDM could be monitored more closely for metabolic complications, and it could guide personalized interventions, such as future deals in diabetes care. Strategies to enhance or restore optimal glycosylation patterns could be explored as adjuncts to traditional GDM management.

## 5. Limitations

The limitations of our study are the small number of cases in the GDM and obese groups, which can be explained by the fact that obesity is so often associated with GDM that it is very difficult to recruit suitable subjects into these groups in the third trimester of pregnancy. The exclusion criteria helped to homogenize the group, but the number of excluded cases was very high.

## 6. Conclusions

By incorporating N-glycan-based biomarkers into clinical diagnosis practice, healthcare providers can rely on early detection, personalize management strategies, and potentially mitigate adverse pregnancy outcomes associated with obesity and GDM.

## Figures and Tables

**Table 1:** Diagnostic criteria for GDM.

Fasting plasma glucose = 5.1–6.9 mmol/L (92–125 mg/dL)
1 h post 75 g oral glucose load ≥ 10.0 mmol/L (180 mg/dL)
2 h post 75 g oral glucose load 8.5–11.0 mmol/L (153–199 mg/dL)

The presence of GDM must be established when at least one of the criteria set by the WHO in 2018 and listed in the table is met [9].

**Table 2:** Demographic characteristics of the groups: normal weight, obese, GDM, and GDM combined with pregnant women with obesity.

Demographical Data	Normal Weight (*n* = 20) (Mean ± SD)	Obese (*n* = 20) (Mean ± SD)	GDM (*n* = 7) (Mean ± SD)	Obese with GDM (*n* = 5) (Mean ± SD)
Maternal age (years)	31.0 ± 4.4	29.0 ± 5.2 ^ns^	34.5 ± 5.15 *	34.0 ± 6.1 *
BMI at the time of first medical visit (kg/m^2^)	23.2 ± 3.1	34.96 ± 4.2 **	24.4 ±.2 ^ns^	41.4 ± 2.7 **
Weight gain during gestation (kg)	14.6 ± 4.6	8.5 ± 5.5 **	8.0 ± 6.3 **	9.0 ± 4.2 **
Weigh gain based on pregestational BMI	normal (11–16 kg)	normal (5–9.5 kg)	normal (5–9.5 kg)	normal (5–9.5 kg)
Gestational age at investigation (weeks)	38.3 ± 1.2	39.4 ± 1.4 ^ns^	37.5 ± 1.9 ^ns^	38.0 ± 2.4 ^ns^

^ns^: non significant, *p* > 0.05, * *p* < 0.05, ** *p* < 0.01 compared to pregnant women with normal body weighted.

**Table 3:** APTS-labeled N-glycan structures identified in serum IgG samples of normal weight, obese, GDM, and obese with GDM mothers..

Peak Number	Structure	GU Value	Normal Weight (Area %)	Obese (Area %)	GDM (Area %)	Obese with GDM (Area %)
1	A2G2S2	4.63 ± 0.01	1.01 ± 0.15	2.02 ± 0.73	2.18 ± 0.75	2.56 ± 1.19
2	A2BG2S2	4.78 ± 0.01	0.49 ± 0.16	0.61 ± 0.28	0.57 ± 0.22	0.70 ± 0.26
3	FA2G2S2	4.91 ± 0.01	2.70 ± 0.16	2.73 ± 0.38	2.61 ± 0.56	2.31 ± 0.66
4	FA2BG2S2	5.01 ± 0.01	1.90 ± 0.08	1.85 ± 0.41	1.71 ± 0.47	1.64 ± 0.47
5	FA2[3]G1S1	5.98 ± 0.11	1.32 ± 0.12	1.68 ± 0.56	1.47 ± 0.33	1.54 ± 0.39
6	A2G2S1	6.20 ± 0.03	1.14 ± 0.08	2.72 ± 0.16	2.04 ± 1.40	2.13 ± 1.19
7	FA2G2S1	6.71 ± 0.07	16.29 ± 0.54	15.67 ± 0.18	12.52 ± 1.58	14.78 ± 4.05
8	FA2BG2S1	6.88 ± 0.03	2.95 ± 0.18	2.53 ± 0.12	2.32 ± 0.71	2.23 ± 0.38
9	FA2; A2[6]G1	7.68 ± 0.22	6.94 ± 0.12	7.63 ± 0.40	11.59 ± 3.29	8.55 ± 4.10
10	FA2B; A2B[6]G1	8.22 ± 0.17	1.54 ± 0.12	1.47 ± 0.11	2.72 ± 0.49	2.21 ± 0.91
11	FA2[6]G1	8.77 ± 0.19	17.71 ± 0.41	17.74 ± 0.74	18.87 ± 1.97	15.94 ± 2.25
12	FA2[3]G1	9.14 ± 0.11	5.16 ± 0.40	5.56 ± 0.35	8.07 ± 1.27	7.21 ± 2.12
13	FA2B[6]G1; A2G2	9.24 ± 0.07	6.38 ± 0.45	5.65 ± 0.26	5.69 ± 1.73	6.74 ± 0.97
14	FA2B[3]G1; A2BG2	9.64 ± 0.07	0.69 ± 0.28	0.68 ± 0.19	1.65 ± 0.44	1.12 ± 0.38
15	FA2G2	10.22 ± 0.02	30.78 ± 0.70	28.85 ± 0.80	23.81 ± 2.36	27.57 ± 5.11
16	FA2BG2	10.57 ± 0.02	2.99 ± 0.21	2.61 ± 0.13	2.18 ± 0.55	2.75 ± 0.25
SF/NF ratio			0.39 ± 0.01	0.43 ± 0.05	0.36 ± 0.09	0.40 ± 0.12
CF/TNG ratio			0.81 ± 0.007	0.79 ± 0.005	0.73 ± 0.030	0.76 ± 0.050

The abbreviated names of the identified N-glycan structures followed the nomenclature proposed by Harvey et al. [15].The calculated relative area percentage values of the detected peaks are given as mean ± SD. SF/NF ratio = ratio of the sialo form to neutral structures; CF/TNG ratio = ratio of core-fucosylated structures to the total N-glycosyaltion.

**Table 4:** Supplement to Figure 2. Comparison of serum IgG N-glycan structures in normal weight (*n* = 20), obese (*n* = 20), GDM (*n* = 7), and obese GDM (*n* = 5) maternal groups using Kruskal–Wallis multiple comparison statistical tests.

Peak Number	Normal Weight vs. Obese	Normal Weight vs. GDM	Normal Weight vs. Obese with GDM	Obese vs. GDM	Obese vs. Obese with GDM	GDM vs. Obese with GDM
1	0.2236	0.0059	0.0005	>0.9999	>0.9999	>0.9999
2	>0.9999	>0.9999	0.2588	>0.9999	>0.9999	0.1201
3	>0.9999	>0.9999	>0.9999	>0.9999	0.7258	0.0151
4	>0.9999	>0.9999	0.9876	>0.9999	>0.9999	>0.9999
5	0.8928	>0.9999	>0.9999	>0.9999	>0.9999	>0.9999
6	0.0208	0.2446	0.2641	0.4287	0.442	>0.9999
7	>0.9999	0.0054	0.7178	0.0318	>0.9999	0.0074
8	0.4103	0.0047	0.0025	>0.9999	>0.9999	>0.9999
9	>0.9999	0.0041	>0.9999	0.0694	>0.9999	<0.0001
10	>0.9999	0.0204	0.7669	0.0089	0.4613	0.0604
11	>0.9999	0.6851	>0.9999	0.6851	>0.9999	<0.0001
12	>0.9999	0.0006	0.0545	0.0082	0.3093	0.1348
13	>0.9999	0.2489	>0.9999	>0.9999	0.2936	<0.0001
14	>0.9999	0.0002	0.2649	0.0001	0.1962	0.0005
15	>0.9999	0.0029	0.8524	0.0193	>0.9999	0.0012
16	0.9754	<0.0001	>0.9999	0.0813	>0.9999	<0.0001
SF/NF	>0.9999	>0.9999	>0.9999	0.6262	>0.9999	0.6496
CF/TNG	>0.9999	<0.0001	0.0247	0.0079	0.4543	0.0520

The difference between the groups was considered significant if *p* ≤ 0.05.

## Data Availability

Data are unavailable to be published due to privacy and ethical restrictions.

## Abbreviations

IgG: immunoglobulin G, DTT: dithiothreitol, SDS: sodium dodecyl sulfate, PNGase F: Peptide-N-Glycosidase F, APTS: 8-aminopyrene-1,3,6-trisulfonic acid, BMI: body mass index, CE-LIF: capillary electrophoresis with laser-induced fluorescent detection, ADCC: antibody-dependent cellular cytotoxicity; Fc: fragment crystallizable; APP: acute phase proteins; RFU: relative fluorescence unit, GDM: Gestational Diabetes Mellitus, SF/NF ratio: ratio of the sialo form to neutral structures; CF/TNG ratio = ratio of core-fucosylated structures to the total N-glycosylation.
